# Research progress on the microbiota in bladder cancer tumors

**DOI:** 10.3389/fcimb.2024.1374944

**Published:** 2024-04-08

**Authors:** Keyuan Lou, Junpeng Chi, Jitao Wu, Jian Ma, Shu Liu, Yuanshan Cui

**Affiliations:** ^1^ Department of Urology, The Affiliated Yantai Yuhuangding Hospital of Qingdao University, Yantai, China; ^2^ Department of Medical Oncology, The Affiliated Yantai Yuhuangding Hospital of Qingdao University, Yantai, China

**Keywords:** bladder cancer, microbiota, review, prognosis, mechanism

## Abstract

The microbiota, also referred to as the microbial community, is a crucial component of the human microenvironment. It is located predominantly in various organs, including the intestines, skin, oral cavity, respiratory tract, and reproductive tract. The microbiota maintains a symbiotic relationship with the human body, influencing physiological and pathological functions to a significant degree. There is increasing evidence linking the microbial flora to human cancers. In contrast to the traditional belief that the urethra and urine of normal individuals are sterile, recent advancements in high-throughput sequencing technology and bacterial cultivation methods have led to the discovery of specific microbial communities in the urethras of healthy individuals. Given the prevalence of bladder cancer (BCa) as a common malignancy of the urinary system, researchers have shifted their focus to exploring the connection between disease development and the unique microbial community within tumors. This shift has led to a deeper investigation into the role of microbiota in the onset, progression, metastasis, prognosis, and potential for early detection of BCa. This article reviews the existing research on the microbiota within BCa tumors and summarizes the findings regarding the roles of different microbes in various aspects of this disease.

## 1 Introduction

Bladder cancer (BCa) ranks among the top ten most common urinary tract malignancies and is the second-most prevalent malignancy of the urinary system. It accounts for 4.6% of all new cancer diagnoses annually, with approximately 400,000 new cases and 160,000 deaths worldwide each year, and has a five-year mortality rate of 30% (Richters et al., 2020). BCa can be divided into muscle-infiltrating bladder cancer (MIBC) and nonmuscle-infiltrating bladder cancer (NMIBC) based on its invasion into the muscular layer of the bladder wall (Lindskrog et al., 2021). Approximately 70% of BCa cases are NMIBC, while the remaining 30% are MIBC, which has a greater potential for invasion and metastasis (Grayson, 2017). The majority of NMIBC patients undergo transurethral resection of the bladder tumor (TURBT), yet the recurrence rates range between 40% and 80%. Additionally, 25% of NMIBC patients progress to MIBC or distant tumor metastases. MIBC, characterized by few early symptoms, rapid progression, and poor prognosis, remains a significant clinical challenge (Antoni et al., 2017).

The incidence of BCa correlates with age, peaking in individuals aged 75-84 years, who represent 30% of new cases annually (Richters et al., 2020). The disease is 3.7 times more common in men than in women (Sung et al., 2021; Yacouba et al., 2022), a disparity attributed to greater exposure to smoking and chemical carcinogens in men, as well as hormonal differences between the sexes (Madeb and Messing, 2004; Yacouba et al., 2022). Despite an increase in smoking among women, the incidence of BCa in this demographic population remains comparatively low, suggesting additional contributing factors beyond established risk factors (Marcon et al., 2018; Yacouba et al., 2022).

Genetic mutations and alterations in specific pathways have been implicated in BCa. Tumor suppressor genes such as TP53, RB1, and PTEN are frequently mutated in carcinoma *in situ* (CIS) (Castillo-Martin et al., 2010). Oncogenes such as FGFR3, PIK3CA, and RAS, which promote tumor cell development, are characteristic of NMIBC (Castillo-Martin et al., 2010; Knowles and Hurst, 2015). Diet, particularly meat consumption, has been reviewed for its role in bladder carcinogenesis due to the formation of carcinogenic chemicals during meat cooking and processing (Aveta et al., 2022).

**Figure 1:**
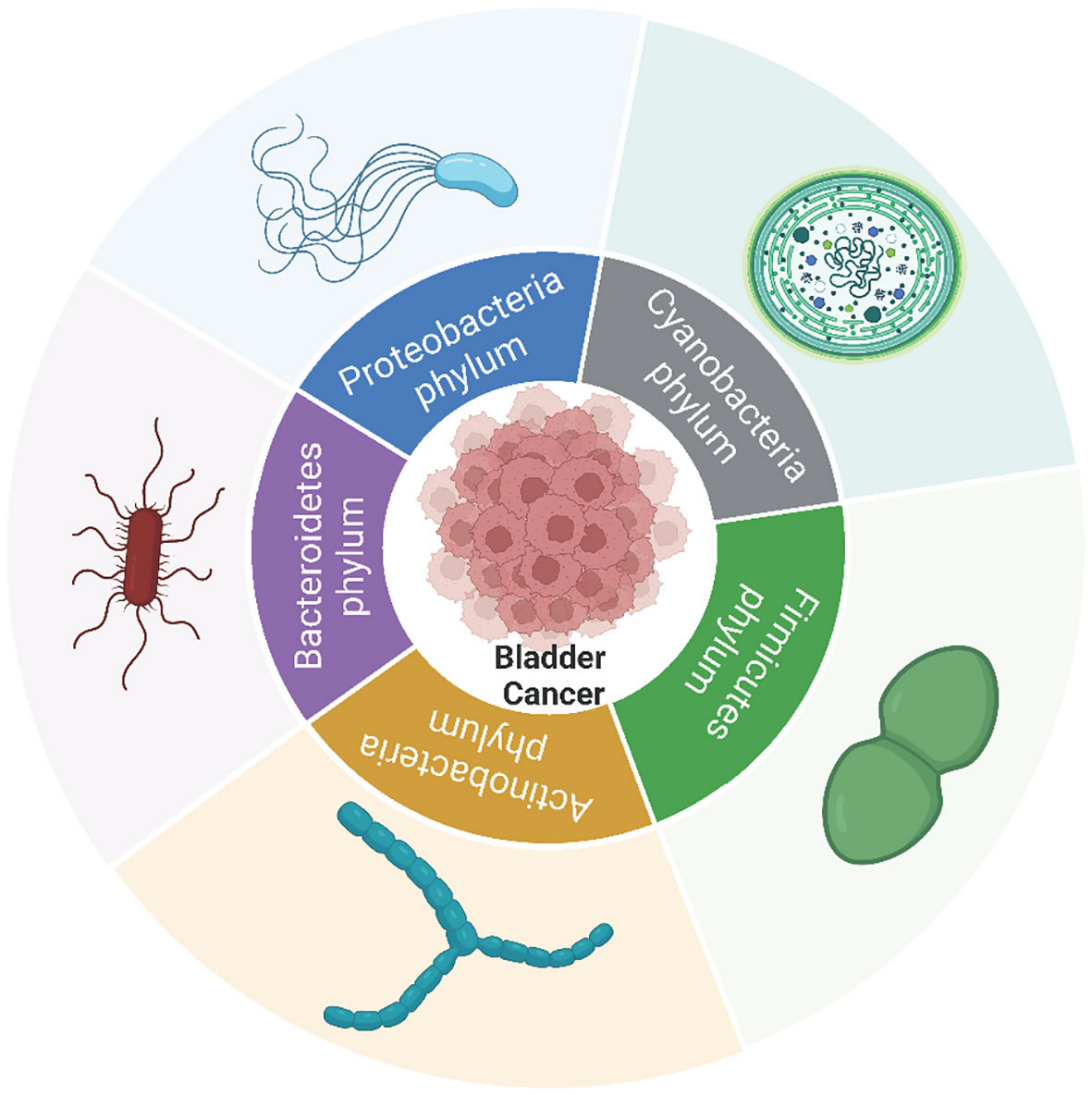
The microbial phyla linked to the onset, progression, metastasis and prognosis of bladder cancer within bladder cancer tumors. Among these, the Proteobacteria, Firmicutes, Bacteroidetes, and Actinobacteria phyla exhibit the highest abundance in bladder cancer as well as adjacent or normal tissues in the majority of studies.

Contrary to previous beliefs that healthy bladders are sterile (Han et al., 2014), advancements in urine collection techniques have revealed a distinct bladder microbiota in healthy individuals (Wolfe et al., 2012; Horwitz et al., 2015; Whiteside et al., 2015a; Thomas-White et al., 2016; Thomas-White et al., 2018). Early research by Hicks RM et al. linked bacteria to schistosomiasis-induced BCa through N-nitrosamine formation (Mansour et al., 2020; Chen et al., 2022). BučevićPopović et al. demonstrated microbial infiltration in 20% of malignant tumor tissues, suggesting bacterial involvement in BCa (Buˇcevi´c Popovi´c et al., 2018). With improvements in detection methods, researchers are increasingly identifying microorganisms in BCa tissues, and exploring their sequencing, functions, and mechanisms. An increasing number of studies indicate that the intratumoral microbiota plays a crucial role in the onset and progression of BCa (**Figure 1**). This article concentrates on various microbial phyla and reviews the diverse roles played by the intratumoral microbiota in BCa.

## 2 Materials and methods

A comprehensive literature search was performed using PubMed, Embase and the China National Knowledge Infrastructure (CNKI) to October 2023. The search terms included ‘bladder cancer tissue’, ‘microbiota’ and ‘microbiome’ (the ‘microbiota’ is defined as the totality of microorganisms living in a specific environment, and the term ‘microbiome’ refers to the totality of microbial genes in a specific biological niche. These terms are often used interchangeably in the literature (Berg et al., 2020). The following criteria were used to include studies in this review: (1) included BCa and control groups (case-control studies), (2) provided information on the presence or abundance of microbial taxa, and (3) provided information on the promotion or inhibition of microorganisms in the BCa and/or control groups. A total of 10 relevant articles were screened, including 8 clinical trial papers and 2 bioinformatics analyses (**Table 1**).

**Table 1:** Characteristics of studies included in the systematic review.

Author	Article type	Population	Stage	Method	High-abundance Microorganisms			Cohorts, Diversity, Abundance and Other Findings
Liu, F.; et al. (Liu et al., 2019)	Original research	22 carcinoma tissues samples (male) and 12 adjacent normal tissues samples	NMIBC 5 MIBC 17	16S rRNA	**Phylum** Proteobacteria(54.1%) Firmicutes(23.7%) Bacteroidetes(13.4%) Actinobacteria (4.4%)	**Genera** *Cupriavidus*(16.9%) *Brucellaceae* (6.0%) *Ralstonia* (5.5%) *Lactobacillus* (5.3%)		1. Verified the occurrence of bladder microbiota dysbiosis in bladder cancer. 2. A statistically significant difference in the Shannon diversity index was observed the abundance of microbiota between cancerous and noncancerous tissues (P = .0417), indicating a significantly lower diversity in cancerous tissues compared to noncancerous tissues. 3. *Lactobacillus* and *Prevotella_9* were enriched in noncancerous tissues, while *Acinetobacter* and *Escherichia‐Shigella* were significantly increased in cancerous tissues. 4. There were no significant differences in patients at different grades and different biologically relevant subtypes with respect to the α‐diversity or β‐diversity‐associated bladder cancer taxa.
Rodriguez, R.M.; et al. (Rodriguez et al., 2020)	Bioinformatics analysis	Total of 56 paired tumor and adjacent normal samples from 28 cases	/	TCGA cancer database	**Phylum** Proteobacteria	**Species** *Stenotrophomonas maltophilia*		1. No statistically significant differences between paired tumor and adjacent normal samples total number of reads, relative abundance, or positivity ratio. 2. In the fully adjusted model, there were no significant differences in alpha diversity. No statistically significant differences were observed in the analyses of paired tumor and adjacent normal samples when stratifying by sex, race, anatomical site, or tumor stage.
Mansour, B.; et al. (Mansour et al., 2020)	Original research	14 tissue samples collected from ten bladder cancer patients (male 5, female 5)	NMIBC 6 MIBC 4	16S rRNA	**Phylum** Firmicutes(34%) Actinobacteria(23%) Proteobacteria(22%) Bacteroidetes(15%) Cyanobacteria(8%)	**Genera** *Bacteroides* *Akkermansia* *Klebsiella* *Clostridium* *Enterobacter*		1. *Akkermansia*, *Bacteroides*, *Clostridium sensu stricto*, *Enterobacter* and *Klebsiella*, referred to as “five suspect genera”, were found to be over-represented in tissue samples compared to the urine. 2. The abundance of microbiota showed significant difference only in the median of genus richness and Shannon diversity between female and male tissue samples.
Pederzoli, F.; et al. (Pederzoli et al., 2020)	Original research	Total of 87 paired urine, neoplastic and nonneoplastic tissues samples from 29 cases (male 21, female 8)	/	16S rDNA	**Genera** *Burkholderia*			1. In tissue samples, no differences were detected in α- and β-diversity between males and females. The genus *Burkholderia* was more abundant in the neoplastic versus the non-neoplastic tissue in both sexes.
Nadler, N.; et al. (Nadler et al., 2021)	Original research	Total 10 BCa samples (male 8, female 2)	MIBC	16S rRNA	/			1. In two (1 male, 1 female) out of ten patients, the analysis showed abundant bacterial aggregation on the surface epithelium, and one sample even indicated what might be considered a submucosal intracellular bacterial community of coccoid bacteria. 2. Interestingly, urothelial cancer tissue from the two patients that exhibited bacterial aggregation did not have a history of positive urine cultures.
Li, W. (Li, 2021)	Original research	Total of 36 paired tumor and adjacent normal samples from 18 cases	/	16S rDNA	**Phylum** Proteobacteria Firmicutes Bacteroidetes Tenericutes			1. Bacteria are present in the outer mucosa, mucosal layer, submucosa, and even the smooth muscle layer of both bladder cancer and tissue adjacent to bladder cancer, with the highest distribution observed in the outer mucosa.. 2. Compared with adjacent tissues, bladder cancer tissues exhibit lower species richness and diversity, but these differences are not statistically significant. Bladder cancer tissues and adjacent tissues have a similar bacterial community structure. 3. At the genus level, the genus with statistical differences in the bladder cancer group is *Staphylococcus*.
Parra-Grande, M.; et al. (Parra-Grande et al., 2022)	Original research	Total of 58 corresponding to 26 patients with paired samples and 6 patients with only tumor tissue samples (male 27, female 5)	MIBC	16S rDNA	**Phylum** Firmicutes (41.46%) Bacteroidetes (28.23%) Proteobacteria (22.78%) Actinobacteria (6.06%)	**Genera** *Bacteroides* (16.24%) *Escherichia.Shigella*(6.07%) *Staphylococcus*(5.43%) *Enterococcus*(4.25%)		1. Significant differences were found in microbial richness at the genus level, with a higher richness observed in the non-tumor mucosa compared to the tumor mucosa (26 samples). Actinobacteria were significantly more enriched in the non-tumor mucosa compared to the tumor mucosa, supporting the hypothesis that a higher abundance of Actinomycetes is associated with a lower rate of bladder cancer in women. 2. In the multivariate analysis, significant differences were found in microbial composition according to tumor grade, as low-grade tumors exhibited a microbial profile that was characterized by a higher enrichment for *Enterococcus*. 3. In the multivariate analysis using the PERMANOVA test, no significant differences were observed between the tumor and non-tumor mucosa regarding microbial composition.
Li, W.T.; et al. (Li et al., 2021)	Bioinformatics analysis	Total 405 BCa samples	MIBC	TCGA legacy archive	/			1. A variety of microbes, including *E. coli*, *butyrate-producing bacterium SM4/1*, and a species of *Oscillatoria*, were associated with expression of classical EMT-associated genes, such as E-cadherin, vimentin, SNAI2, SNAI3, and TWIST1. 2. There are significant correlations between microbial abundance and the expression of genes in the ECM, specifically collagens and elastin.
Mansour, B.; et al. (Mansour et al., 2022)	Original research	55 bladder cancer tissue samples and 12 prostatic hyperplasia tissue samples as control	/	16S rRNA	**Phylum** Firmicutes(46%) Proteobacteria(23%) Actinobacteria(13%) Bacteroidetes(11%)			1. Pronounced differences were observed in both alpha and beta microbiome diversity between the tumor (bladder cancer) and non-tumor (prostatic hyperplasia) tissue samples. 2. The microbiome β‐diversity of the 32 male and 14 female tumor samples also showed significant differences. 3. The combined increase in urine HBD2 and HBD3 levels reduces the abundance of non-tumor specific genera (*Bacteroides*, *Parabacteroides*, *Faecalibacterium*) and increases the abundance of more common in-tumor tissue genera (*Staphylococcus*, *Corynebacterium*).
Sun, J.X.; et al. (Sun et al., 2023)	Original research	Total 22 BCa samples (mainly male)	NMIBC 7 MIBC 15	2bRAD-M	**MIBC**			1. The microbial diversity of NMIBC tissues was significantly higher than that in MIBC tissues. The microbial composition of the two tumor tissues was similar, with *Ralstonia_sp000620465* was the most dominant species. 2. Functional annotation analysis showed 3011 different COGs and 344 related signaling pathways between MIBC and NMIBC microbiomes.
					**Phylum** Proteobacteria(68.45%) Firmicutes (14.76%) Actinobacteriota (12.98%)	**Genera** *Ralstonia* (56.29%) *Cutibacterium* (9.82%) *Enterococcus* (6.91%) *Sphingomonas* (5.77%) *Metamycoplasma*(4.60%)	**Species** *Ralstonia_mannitolilytica* *Ralstonia_pickettii* *Ralstonia_sp000620465*	
					**NMIBC**			
					**Phylum** Proteobacteria(39.09%) Firmicutes (19.17%) Actinobacteriota (14.92%) Firmicutes_A (13.13%) Bacteroidota (11.55%)	**Genera** *Ralstonia* (22.16%) *Cutibacterium* (6.60%) *Bacteroides* (5.51%) *Staphylococcus* (5.27%) *Acinetobacter* (5.07%)	**Species** *Acinetobacter_guillouiae* *Anoxybacillus_A_rupiensis* *Brevibacillus_agri* *Staphylococcus_lugdunensis*	

## 3 Phylum Proteobacteria

Using data from the TCGA database, Rebecca M. Rodriguez et al. analyzed microbial diversity and species differences in 56 paired tumor and adjacent normal tissue samples from 28 patients. They discovered that Proteobacteria was the most prevalent phylum, comprising 93% of the total reads, with *Stenotrophomonas maltophilia* being the most common species (accounting for 61% of the total reads) (Rodriguez et al., 2020). Wei Li conducted genomic sequencing of bacterial species in cancer and adjacent tissues from 18 BCa patients without urinary tract infections and with negative urine cultures. This study corroborated the existence of microbial flora in bladder tissue, revealing that the most abundant bacteria in both cancer and adjacent bladder tissues belonged to the phylum Proteobacteria (Li, 2021). Jian-Xuan Sun’s team employed 2bRAD-M microbiome sequencing technology to analyze tumor tissue samples from 22 BCa patients, focusing on the differences in the microbial community between NMIBC and MIBC. Echoing the findings of previous studies, they noted similar microbial compositions in both tumor types, with *Ralstonia_sp000620465* being the predominant species (Sun et al., 2023).

Proteobacteria are currently the largest phylum within the domain Bacteria. A common trait of Proteobacteria is the gram-negative staining and, thus, the presence of lipopolysaccharide in the outer membrane. Many common human pathogens are found in the Proteobacteria phylum, such as *Brucella* genera, *Rickettsia* genera, *Neisseria*, *Escherichia*, *Shigella*, *Salmonella* and *Helicobacter* genera (Human Microbiome Project Consortium, 2012). Proteobacteria, which are typically gut commensals with pathogenic potential, have been studied extensively (Everard et al., 2011; Geurts et al., 2011; Zhang et al., 2012). Recent research highlighted significant variations in the abundance of Proteobacteria in mucosa-associated microbiomes of ileal and rectal biopsies (but not in stool samples) between Crohn’s disease patients and control subjects (Gevers et al., 2014). Similar shifts in gut proteobacterial communities have been observed in patients with colitis-associated colorectal cancer (Bonnet et al., 2014). These findings collectively suggest that an imbalance in Proteobacteria could play a crucial role in bladder carcinogenesis. The increase in the abundance of Proteobacteria might serve as a potential diagnostic indicator for dysbiosis, potentially increasing the risk of BCa.

### 3.1 *Cupriavidus* in BCa

Fei Liu et al. examined tissue samples of cancerous bladder mucosa from patients diagnosed with BCa, consisting of 22 carcinoma tissues and 12 adjacent normal tissues. This study confirmed the occurrence of bladder microbiota dysbiosis in BCa patients. The researchers observed that Proteobacteria was the predominant phylum in both cancerous and noncancerous tissues, with a significantly greater abundance in cancerous tissues. In cancerous tissues, the presence of *Cupriavidus*, *Acinetobacter*, and *Escherichia‐Shigella* increases markedly (Wang et al., 2007). Previous research has indicated that certain trivalent pesticide-related chemicals can induce protein carbonylation and oxidative DNA damage in human urothelial cells, potentially leading to BCa (Liu et al., 2019). Interestingly, the enrichment of harmful chemical products, which are subject to metabolic processes, might correlate with the significantly elevated abundance of the genus *Cupriavidus*. This genus was the most abundant bacteria found in both cancerous and noncancerous bladder tissues, despite notable differences in their levels. *Cupriavidus*, known from prior studies as an organophosphorus pesticide-degrading microorganism, is thought to break down harmful substances absorbed by the body, subsequently excreting them into the bladder through specific enzymes (Wang et al., 2007).

### 3.2 Biofilms in BCa development

Naomi Nadler et al. collected tissue samples from ten patients undergoing TURBT and analyzed them for bacterial aggregates using Fluorescence *in situ* Hybridization (FISH). Dense biofilms were identified in the urothelial cancer tissue of two samples, and spherical bacteria were confirmed in one sample. Notably, both patients had negative preoperative urine cultures. These findings suggest a potential link between biofilm formation and BCa (Nadler et al., 2021).

Bacterial aggregates, commonly referred to as biofilms, often attach to the apical epithelial cells of the bladder and are known as umbrella cells. These cells typically have a protective layer of sulfated polysaccharide aminoglycans. Disruption of this layer may lead to pathological bladder changes and chronic inflammation in the genitourinary system (Garrett, 2015). During biofilm formation, bacteria such as *Aeromonas hydrophila* and *Pseudomonas aeruginosa* can augment the production of extracellular proteases, including serine proteases, metalloproteinases, and elastases (Swift et al., 1999). Some pathogens, such as *Streptococcus pyogenes*, may cause invasive diseases by degrading intercellular junctions in conjunction with the host cysteine protease calpain (Sumitomo et al., 2013).

Studies on bacterial biofilms in colorectal cancer have shown that certain bacteria, such as *Escherichia coli*, can drive cancer development. *Escherichia coli* produces the genotoxin colibactin, which causes mutagenic DNA damage through covalent DNA binding to a chemical moiety known to promote tumorigenesis (Garrett, 2015). Further research is required to understand this relationship, particularly whether bacterial dysbiosis and biofilm formation in the bladder exhibit oncogenic features similar to those in colorectal cancer. Establishing a causal link could make microbiome modulation a significant therapeutic area.

### 3.3 Microbial associations with the epithelial-mesenchymal transition in BCa development

Wei Tse Li et al. identified a range of microbes, including *E. coli*, the butyrate-producing bacterium SM4/1, and a species of *Oscillatoria*, that were correlated with the expression of classic EMT-associated genes, such as E-cadherin, vimentin, SNAI2, SNAI3, and TWIST1. Notably, the *Escherichia coli str. K-12 substr.* showed the most significant correlations with EMT-related gene expression. They also observed significant correlations between microbial abundance and the expression of extracellular matrix (ECM) genes, particularly those related to collagens and elastin. The presence of the *Escherichia coli O157:H7* strain exhibited a significant correlation with the expression of ECM proteins (Li et al., 2021).

EMT is a key process in several cancers that enhances metastatic potential by increasing cell mobility and decreasing cell–cell adhesion (Franzen et al., 2015; Ogawa et al., 2020). During EMT, epithelial cells transform into mesenchymal cells, enabling detachment from the basement membrane and invasion into adjacent tissues. These mesenchymal cells can migrate to distant sites and revert back to epithelial cells through mesenchymal–epithelial transition, initiating metastasis. In MIBC, the importance of EMT is highlighted by the upregulation of mesenchymal cell markers, such as N-cadherin and P-cadherin, and the downregulation of epithelial cell markers, such as E-cadherin (Yun and Kim, 2013; Franzen et al., 2015). ECM proteins play a vital role in cancer cell invasion and metastasis, with proteins such as collagens, laminins, and fibronectins associated with survival in urothelial bladder cancer (Brunner and Tzankov, 2007). During metastasis and invasion, ECM proteins degrade and integrins rearrange, facilitating EMT (Brunner and Tzankov, 2007; Jiang et al., 2017). Microbes, known for releasing proteases including collagenases, are thought to influence ECM protein turnover, although this has not yet been confirmed in BCa studies (Alfano et al., 2016).

Interactions between tumor cells and the extracellular microenvironment are critical in cancer development and progression. The interplay between ECM components and bacterial products regulates tissue homeostasis, dysregulation of these processes may create protumorigenic niches, potentially contributing to disease relapse. Understanding the relationship between *Escherichia coli* and the extracellular microenvironment offers valuable insights into the pathogenesis and progression of BCa.

### 3.4 Role of *Burkholderia* in BCa pathobiology

Filippo Pederzoli and his team conducted a study on paired samples from 21 male and 8 female patients, including urine, neoplastic (Npl), and non-neoplastic (non-Npl) tissues. Their findings revealed a greater abundance of the genus *Burkholderia* in tumor tissues than in non-tumor tissues, regardless of the patient’s gender (Pederzoli et al., 2020). In a parallel study, Bassel Mansour’s research group corroborated these results. They investigated microbiome differences in urine and tissue samples from BCa patients and noted a greater abundance of *Burkholderia* in cancerous tissues (Mansour et al., 2020). This observation implies a potential role for this genus in the pathobiology of BCa, similar to recent findings in colorectal cancer versus healthy colon mucosa (Alomair et al., 2018). However, the role of this taxon appears to extend beyond merely acting as a trigger for neoplasia. Recent studies have suggested *Burkholderiales* as a potential “anticancer probiotic.” For instance, in an animal sarcoma model, it was demonstrated that the efficacy of immunotherapy with CTLA-4 antibody was influenced by the composition of the microbiota, particularly by *Bacteroides fragilis*, *Bacteroides thetaiotaomicron*, and *Burkholderiales* (Vétizou et al., 2015). Additionally, the transplantation of these bacteria into antibiotic-conditioned animals showed a protective effect against colitis induced by CTLA-4 blockade.

## 4 Phylum Cyanobacteria

Cyanobacteria, also known as blue-green algae, are an ancient, highly diverse group of photoautotrophic organisms, that evolve a wide variety of morphologies reaching from unicellular to filamentous organization and thereby represent one of the most diverse prokaryotic phyla. In the past, they were frequently grouped under algae; however, Cyanobacteria exhibited significant distinctions from eukaryotic organisms. Devoid nuclear membranes and organelles and, with their genetic material, DNA that is not organized into chromosomes are characteristic features of bacteria. Consequently, they are now classified within the domain Bacteria (Schirrmeister et al., 2011). Cyanobacteria are considered the inventors of oxygenic photosynthesis, they employ photosynthetic pigments such as carotenoids, phycobilins, and various forms of chlorophyll to capture light energy (Sagan, 1967). Apart from performing oxygenic photosynthesis, many cyanobacteria are able to fix atmospheric nitrogen, adding to their importance in natural ecosystems (Welsh et al., 2008). Cyanobacteria serve as important model organisms with potential biotechnological applications. They are utilized in bioethanol production, edible pigments, human and animal food, nutritional supplements, and raw materials (Tan, 2007; Demay et al., 2019). Cyanobacteria are also known to produce various toxins, termed cyanotoxins, which may pose risks to humans and animals (van Apeldoorn et al., 2007).

### 4.1 *Oscillatoria* and EMT-associated gene expression

Wei Tse Li et al. discovered a strong negative correlation between the presence of *Oscillatoria*, a Cyanobacteria member, and the expression of EMT-promoting genes (Li et al., 2021). Although Cyanobacteria are less characterized in humans, their presence has been detected in the gut (Almeida et al., 2019). Importantly, *Oscillatoria* species produce butylated hydroxytoluene, a natural antioxidant, which might explain its association with reduced EMT (Babu and Wu, 2008). This connection is significant as oxidative stress and reactive oxygen species are known to regulate ECM proteins and the EMT process (Jiang et al., 2017).

### 4.2 Cyanobacteria in BCa: microbiota composition and human β-defensins response

Bassel Mansour et al. compared the microbiota composition in cancerous tissues and urine samples from the same patient set. They noted a surprisingly high presence of Cyanobacteria—7% and 8% in urine and tissue samples, respectively—a finding previously undocumented (Mansour et al., 2020).

Mansour, Ádám Monyók, and their team subsequently investigated the tissue microbiome in BCa patients compared to that in benign prostatic hyperplasia patients and healthy volunteers. The authors focused on the mRNA levels of hBDs in tissue and the levels of defensin in urine. *Oxyphotobacteria*, a Cyanobacterium genus linked to tumor formation (Miao et al., 2016), exhibited a significantly greater abundance in the BCa group, especially in patients with low urinary hBD-1 levels (Mansour et al., 2022).

Defensins, notably hBDs, are critical antimicrobial peptides that play a role in tumor cell dissolution, immune cell attraction, and interactions with complement factors (Adyns et al., 2023). Human β-Defensin-1 (hBD-1) can modify HER2 signal transduction and has been shown to suppress BCa growth (Sun et al., 2019). The ability of hBD-1 to attract cells expressing C-C chemokine receptor 6 (CCR6) suggests its potential for recruiting immune cells, such as Th cells, Tregs, imDCs, and neutrophils (Yang et al., 1999; Biragyn et al., 2001; Wu et al., 2003). The dual antitumor and antibacterial activities of hBD-1, along with the influence of bacterial presence on the production of hBD-2 and hBD-3 (Adyns et al., 2023), highlight the significance of understanding the role of hBDs in antitumoral immune responses and their potential impact on immunotherapy effectiveness.

## 5 Phylum Firmicutes

Generally, the phylum Firmicutes is recognized for comprising low G + C gram-positive microorganisms characterized by rigid or semi-rigid cell walls containing peptidoglycan. The cells of Firmicutes microorganisms typically take the form of rods or spheres and primarily reproduce through binary fission. Some members can form endospores and exhibit motility facilitated by flagella (Seong et al., 2018). Firmicutes are abundant in soil and aquatic environments and play a crucial role in the decomposition and recycling of organic matter (Baik et al., 2008). Nevertheless, certain genera within this phylum constitute normal flora in the mammalian intestine or act as pathogens for humans, animals, and plants (Lee et al., 2009; Nguyen and Götz, 2016). Additionally, some members of the Firmicutes phylum hold industrial significance for their role in the production of antibiotics, enzymes, and dairy products (Kwak et al., [[NoYear]]; Liu et al., 2012).

### 5.1 Lactic acid bacteria (LAB) in BCa

LAB, a group of gram-positive bacteria within the phylum Firmicutes, are primarily categorized into the genera *Lactobacillus*, *Streptococcus* and *Lactococcus* (Sharma et al., 2020).

Fei Liu’s research indicated a notable difference in the abundance of *Lactobacillus* between cancerous and noncancerous tissues, with a greater prevalence in noncancerous tissues (Wang et al., 2007). *Lactobacillus*, a well-studied probiotic, is known for its health-promoting mechanisms, such as colonizing resistance, acid production, and pathogen exclusion. It has also been linked to cancer, as cell-free supernatants from *Lactobacillus* can reduce the invasion ability of metastatic tumor cells *in vitro* (Siddiqui et al., 2012; Pearce et al., 2014). The predominant strain *Lactobacillus* in women’s bladders and its potential protective role against urothelial bladder cancer have not been determined, although there is evidence suggesting its role in reducing chronic inflammation and enhancing immune responses (Kato et al., 1984; Kato et al., 1988; Takagi et al., 2001; Matsumoto et al., 2009; Shida and Nomoto, 2013).

### 5.2 *Staphylococcus* and its relationship with BCa

Wei Li’s study sequenced and analyzed bacterial genomes from cancer and adjacent tissues of 18 BCa patients with no urinary tract infection and negative urine culture. The research has revealed the presence of bacteria in various bladder layers, predominantly in the outer layer of the mucosa. Linear discriminant analysis Effect Size (LEfSe) revealed significant differences in microbial communities, highlighting *Staphylococcus* at the genus level as significantly more abundant in BCa tissues. This finding aligns with multiple cases of *Staphylococcus* in the urine cultures of BCa patients (Li, 2021). Similarly, Bassel Mansour’s team noted a greater abundance of *Staphylococcus* in tumor tissues, correlating with elevated levels of hBD-2 and hBD-3 in urine (Mansour et al., 2022). The inducible production of antibacterial HBD2 and HBD3 is affected by bacteria. Elevated levels of HBD2 were shown to cause treatment failure in anticancer immunotherapy (Adyns et al., 2023). *Staphylococcus*, particularly saprophytic *Staphylococcus*, is commonly associated with urinary tract infections. Its role in chronic infections and potential link to the development of BCa warrant further investigation. The impact of saprophytic *Staphylococcus* on bladder epithelial and cancer cells could be a valuable direction for future research.

## 6 Phylum Actinobacteria

In terms of the number and variety of identified species, the phylum Actinobacteria represents one of the largest taxonomic units among the 18 major lineages currently recognized within the domain Bacteria (Stackebrandt et al., 1997). Actinobacteria display a wide variety of morphologies, ranging from coccoid or rod-coccoid to fragmenting hyphal forms or permanent and highly differentiated branched mycelia. They also exhibit diverse physiological and metabolic properties, including the production of extracellular enzymes and the formation of a wide variety of secondary metabolites. Many of these secondary metabolites are potent antibiotics (Ventura et al., 2007).

### 6.1 Actinobacteria: diverse roles in BCa dynamics

Since 1976, BCG (*Mycobacterium bovis*, Bacillus Calmette–Guérin) has been used as one of the most successful antitumor immunotherapies, particularly for the treatment of BCa (Morales et al., 2017). This specific strain from the Actinobacteria phylum and *Mycobacterium* genus not only activates the immune system against BCa but also directly induces tumor cell apoptosis (Yu et al., 2015), necrosis (See et al., 2009), or oxidative stress (Shah et al., 2014). The lower incidence of BCa in women might be partially explained by the significantly greater presence of Actinomycetes, including the *Mycobacterium* genus, in the female urinary microbiome (Lewis et al., 2013).

Mónica Parra-Grande et al. observed a significantly greater abundance of Actinobacteria in nonneoplastic bladder mucosa than in tumor tissues (P = 0.014). Although not statistically significant at the genus level, *Propionibacterium* was more abundant in the non-tumor mucosa (P = 0.08) (Parra-Grande et al., 2022). *Propionibacterium freudenreichii*, notable for its probiotic potential and commercial relevance, has been suggested to be a protective agent against colorectal cancer. Research by Casanova et al. indicated that this bacterium could be useful as a probiotic for early-stage colorectal cancer prevention (Casanova et al., 2018). This finding supports the hypothesis that a microbiota rich in Actinomycetes might be linked to the lower incidence of BCa in women (Raoult, 2017), suggesting that a preventive effect similar to that of the BCG vaccine (composed of Actinomycetes), known for its protective role in BCa treatment and relapse prevention (Whiteside et al., 2015b).

Conversely, the research team led by Bassel Mansour and Ádám Monyók reported a greater abundance of another Actinobacteria phylum member, the *Corynebacterium* genus, in tumor specimens than in non-tumor specimens. This increased presence correlated with elevated levels of hBD-2 and hBD-3 in the urine (Mansour et al., 2022), suggesting a possible role of *Corynebacterium* in BCa pathogenesis.

### 6.2 Health-promoting bacterium: *Bifidobacterium* in BCa

The *Bifidobacterium* genus is a strictly anaerobic, gram-positive, pleomorphic rod-shaped bacterium that belongs to the Actinobacteria phylum. Owing to their morphological and physiological similarities with *Lactobacillus*, they were historically classified as members of the *Lactobacillus* genus for a significant portion of the 20th century. Only recently have they been acknowledged as a distinct genus separate from *Lactobacillus* (Turroni et al., 2011). Many bifidobacteria are used as active ingredients in a variety of so-called functional foods due to their perceived health-promoting or probiotic properties, such as protection against pathogens mediated through the process of competitive exclusion, bile salt hydrolase activity, immune modulation, and the ability to adhere to mucus or the intestinal epithelium (Liévin et al., 2000; Ouwehand et al., 2002; Stanton et al., 2005).

Mónica Parra-Grande et al. reported an increased abundance of *Bifidobacterium* in NMIBC tissues (Whiteside et al., 2015b). *Bifidobacterium* plays a crucial role in microbial homeostasis and anti-inflammatory responses in the mucosa (Di Giacinto et al., 2005; Turroni et al., 2011), and can inhibit IL-6 secretion (Trikha et al., 2003), indicating its possible influence on tumorigenesis. Vyara Matson’s study revealed a link between enriched *Bifidobacterium* and improved immunotherapy response in melanoma patients (Morales et al., 2017). Parra-Grande’s findings suggest that a lower abundance of *Bifidobacterium* in MIBC than in NMIBC could indicate bladder mucosal damage and a protective factor against BCa (Parra-Grande et al., 2022).

## 7 Phylum Bacteroidetes

The phylum Bacteroidetes consists of more than 7000 different gram-negative bacteria, primarily falling within the genera *Bacteroides*, *Prevotella*, *Parabacteroides*, and *Porphyromonas* (Wexler, 2007). Bacteroidetes, particularly species within the *Bacteroides* and *Prevotella* genera, are major degraders of complex carbohydrates, and possess a variety of polysaccharide and glycoside hydrolases capable of breaking down polysaccharides. They play a crucial role in the breakdown of dietary fiber and starch, release energy, and may act as a primary source of propionate (Tuson et al., 2018). Bacteroidetes are actively involved in immunomodulation, with certain members contributing to the suppression of inflammatory activities, while others may promote inflammation, and some are recognized as opportunistic pathogens. Furthermore, the phylum Bacteroidetes plays a role in the regulation of metabolic syndrome and the gut-brain-axis, with intriguing therapeutic implications for mood impairment and neurologic disorders (Gibiino et al., 2018).

### 7.1 Implications of *Prevotella_9* for cancer research

In their study, Fei Liu et al. reported that *Prevotella_9* was more prevalent in non-cancerous tissues (Wang et al., 2007). *Prevotella*, an integral part of the gut microbiome, plays a crucial role in the digestion of fiber and complex carbohydrates. Its abundance is closely linked to dietary habits, particularly when it is high in fiber. Members of the *Prevotella* genus have been implicated in various cancers, including colorectal, oral, and stomach cancers (Feng et al., 2023). For instance, an increase in *Prevotella* has been noted in the gut microbiome of colorectal cancer patients (Bonnet et al., 2014). Some *Prevotella* species may contribute to cancer development by affecting the tumor microenvironment, for example, through modulating local immune responses or producing metabolic byproducts. There is also evidence suggesting that *Prevotella* may impact the host immune system, potentially playing a role in tumor immune evasion (Chen et al., 2022). While the general research on *Prevotella* highlights its potential significance in cancer development, additional studies are needed to elucidate the specific role and mechanisms of *Prevotella_9* in cancer. The greater abundance of *Prevotella_9* in non-cancerous tissues, as found by Fei Liu, might indicate distinct characteristics and roles of this subtype within the *Prevotella* genus.

## 8 Others

### 8.1 Diversity and discrepancies in BCa microbiota studies

Several studies included in this summary have conducted comparative analyses of BCa tissues, adjacent normal tissues, or non-tumor tissue samples. Bassel Mansour’s team highlighted significant differences in microbial composition between tumor and non-tumor tissue samples (Mansour et al., 2022). Fei Liu’s team found that, compared with noncancerous tissues, cancerous tissues had lower species richness and diversity, with notable differences in beta-diversity (Wang et al., 2007). Similarly, Mónica Parra-Grande’s team observed greater microbial diversity in non-tumor bladder mucosa than in tumor tissues, aligning with global indicators of microbiome diversity and richness (Parra-Grande et al., 2022). However, Rebecca M. Rodriguez’s team reported no significant statistical differences in total reads, relative abundance, or positivity ratio between paired tumor and adjacent normal samples (Rodriguez et al., 2020), a finding echoed by Wei Li et al (Li, 2021).

Regarding microbiota variations in BCa tissues of different stages and grades, Mónica Parra-Grande’s team noted significant differences in microbial composition among different tumor grades (Parra-Grande et al., 2022). Jian-Xuan Sun’s team reported similar microbial compositions in MIBC and NMIBC tumor tissues, but with notably greater microbial diversity in NMIBC tissues (Sun et al., 2023). These findings suggest a potential link between the microbiome and tumor biology. Conversely, Bassel Mansour et al. observed no differences in beta-diversity across different tumor grades and stages or in relation to diabetes and hypertension (Mansour et al., 2022). Similarly, Rebecca M. Rodriguez et al. reported no significant differences when paired tumor and normal tissues were stratified according to sex, race, anatomical site, or tumor stage (Rodriguez et al., 2020).

In the context of sex differences, Bassel Mansour et al.’s studies indicated significant diversity variations between male and female tissue samples (Mansour et al., 2020; Mansour et al., 2022), whereas Filippo Pederzoli et al.’s research showed no differences in alpha- and beta-diversity based on sex (Pederzoli et al., 2020). The study of the BCa tumor microbiota is a burgeoning field, with discrepancies among studies highlighting the need for larger sample sizes and further research.

## 9 Discussion

By reviewing the current research on the intratumoral microbiota of BCa, we identified potential mechanisms through which microorganisms can impact the onset, progression, and prognosis of this disease. Possible mechanisms that may promote Bca include: *Cupriavidus*, which may lead to the enrichment of metabolizable harmful chemical products within the bladder, inducing protein carbonylation and oxidative DNA damage in human urothelial cells. Bioflms may augment the production of extracellular proteases, degrade intercellular junctions in conjunction and cause mutagenic DNA damage. *Escherichia coli* may exhibit a significant correlation with the expression of EMT-related genes and ECM proteins. *Burkholderi* may play a role contrary to other studies where it acts as an “anticancer probiotic” affecting the immune therapeutic effects of CTLA-4 antibodies. *Oxyphotobacteria* may influence the levels of hBD-1 in urine, affecting its anticancer effect on the HER2 signaling pathway and its antimicrobial effect on recruiting immune cells. *Corynebacterium* and *Staphylococcus* may be associated with the elevated levels of hBD-2 in urine, leading to failure of anticancer immunotherapy. Besides, *Staphylococcus* may play a potential role in chronic urinary tract infections, increasing the risk of bladder cancer.

**Figure 2:**
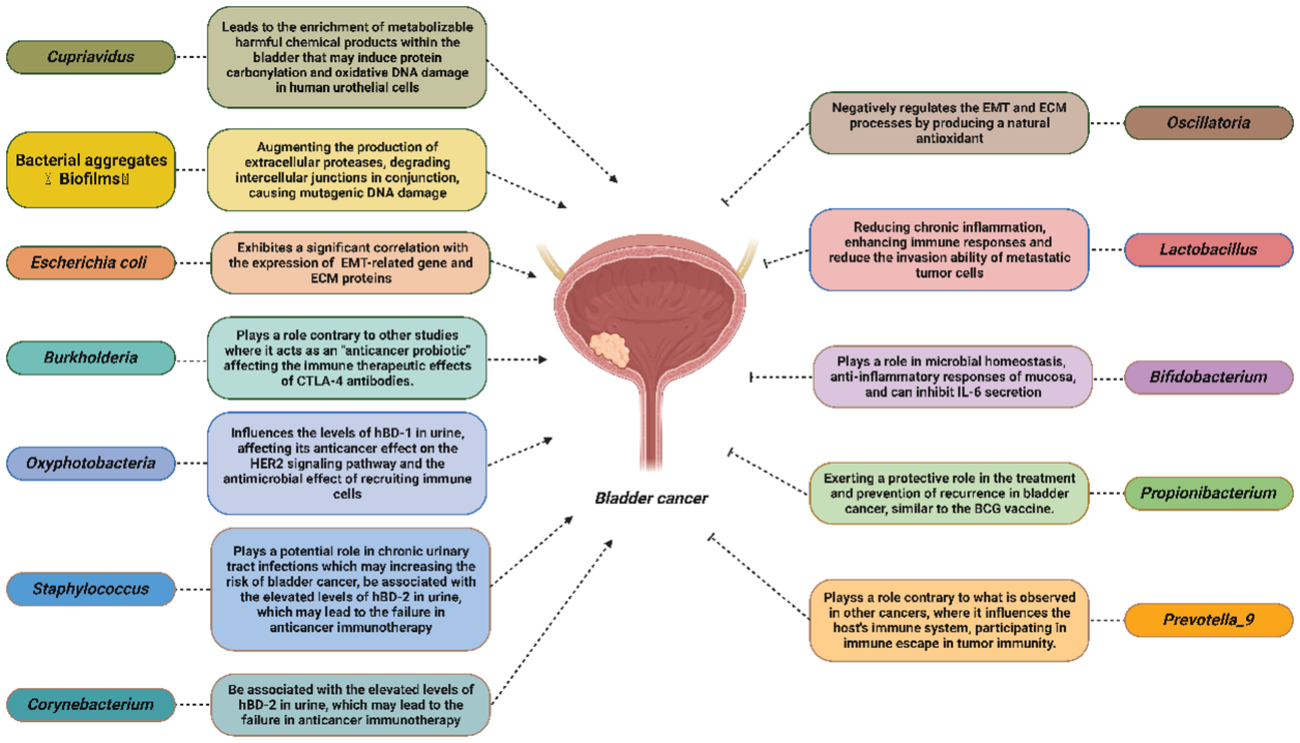
Potential mechanisms of different intratumoral microbiota in the onset, progression, metastasis, prognosis and other aspects of bladder cancer.

Moreover, there are potential anticancer mechanisms, for example, *Oscillatoria* may negatively regulate the EMT and ECM processes by producing a natural antioxidant. *Lactobacillus* may reduce chronic inflammation, enhance immune responses and reduce the invasion ability of metastatic tumor cells. *Bifdobacterium* may play a role in microbial homeostasis, anti-inflammatory responses of the mucosa, and may inhibit IL-6 secretion. *Propionibacterium* may exert a protective role in the treatment and prevention of bladder cancer recurrence, similar to the effects of the BCG vaccine. *Prevotella_9* may play a role contrary to what is observed in other cancers, where it influences the host immune system and participates in immune escape in tumor immunity (**Figure 2**).

Simultaneously, we have contemplated several questions: How do microorganisms enter BCa tumors and in what form? What role does the intratumoral microbiota play in antitumor immunity? Does it have a positive, negative, or surveillance effect, and to what extent is it an instigator, collaborator, or passive observer? Is the intratumoral microbiota detected in BCa a result of purposeful selection by the human body? Does the intratumoral microbiota influence the neural innervation of BCa tumors? What is the extent of the association between the intratumoral microbiota and the recurrence and prognosis of BCa? What kinds of interactions occur among different intratumoral microbiota groups within the tumor? These unresolved questions may lead to new research ideas and directions in the future.

Furthermore, we have identified several limitations that impede research progress. First, ethical considerations prevent the use of healthy samples as controls, thereby hindering comprehensive comparisons. Second, many studies often feature small sample sizes, and lack strong representativeness of the broader population. Third, ensuring complete sample contamination-free processes, such as collection, transportation, storage, and detection, poses a significant challenge, as contamination can potentially impact experimental results. Fourth, different surgical procedures may introduce intergroup differences. Fifth, the small volume of tumor tissue, low bacterial biomass within the tumor, and uncultivable characteristics of some microbial species increase the difficulty of detection. Moreover, tumor samples exhibit a very high host-to-bacterial DNA ratio, potentially causing bias in amplicon-based sequencing results. Additionally, the intrinsic limitations of amplicon-based microbiome methods and incomplete reference databases decrease the extrapolation reliability of these methods. Patient-specific factors such as age, race, sex, breastfeeding status, diet, socioeconomic status, epidemiological factors, genetics, exposure to environmental carcinogens, or disease-specific factors such as pathological TNM stage and focality remain uncontrollable variables. Finally, potential bias in samples introduced by previous BCa diagnostic procedures (e.g., cystoscopy) or prior antibiotic therapies (>4 weeks) cannot be excluded, although the impact of these procedures on the bladder and urinary microbiome is currently unclear.

There are still significant gaps in clinical and laboratory research on the intratumoral microbiota of BCa, and there are several contradictory conclusions among different studies. Addressing these challenges and undertaking innovative or in-depth explorations in this field remains a long journey for researchers. In the future, delving into the molecular mechanisms of the microbiota’s role in BCa tumors, identifying new treatment targets and biomarkers, and exploring how to manipulate the BCa intratumoral bacterial community for cancer patient treatment are crucial. This finding suggests that the biological contribution of the microbiota to BCa is likely to occupy an increasingly prominent position in future BCa research.

## 10 Conclusions

In conclusion, this article reviews the existing research on the microbiota within BCa tumors, summarizes the findings regarding the roles of different microbes in various aspects of this disease and reflects on the current challenges and future research directions in this field. It is hoped that this study will provide effective assistance for a better understanding of BCa and offer some ideas for further innovative development of the field of intratumoral microbiota in BCa.

## Author contributions

KL: Data curation, Formal analysis, Software, Writing – original draft. JC: Methodology, Software, Writing – original draft. JW: Investigation, Methodology, Writing – original draft. JM: Project administration, Resources, Writing – original draft. SL: Supervision, Validation, Writing – original draft. YC: Conceptualization, Data curation, Formal analysis, Funding acquisition, Writing – review & editing.
